# Draft Genome Sequence of Rhodococcus aetherivorans JCM 14343^T^, a Bacterium Capable of Degrading Recalcitrant Noncyclic and Cyclic Ethers

**DOI:** 10.1128/MRA.01345-19

**Published:** 2020-01-16

**Authors:** Daisuke Inoue, Masatoshi Nakazawa, Norifumi Yamamoto, Kazunari Sei, Michihiko Ike

**Affiliations:** a Division of Sustainable Energy and Environmental Engineering, Osaka University, Suita, Osaka, Japan; b Technology Center, Taisei Corporation, Yokohama, Kanagawa, Japan; c Department of Health Science, Kitasato University, Sagamihara-Minami, Kanagawa, Japan; Loyola University Chicago

## Abstract

Here, we report the draft genome sequence of Rhodococcus aetherivorans JCM 14343^T^, which possesses the versatile ability to degrade recalcitrant noncyclic and cyclic ether compounds. The 4.2-Mbp genome of this bacterium contains alkane hydroxylase and propane monooxygenase genes involved in the degradation of noncyclic and cyclic ethers, respectively.

## ANNOUNCEMENT

Members of the genus *Rhodococcus* possess the versatile ability to catabolize a variety of xenobiotic organic pollutants and are useful for the bioremediation of polluted environments (1, 2). Among the members of this genus, Rhodococcus aetherivorans is a species of special interest in the biodegradation of various recalcitrant organic pollutants, such as noncyclic and cyclic ethers (3–5), petroleum compounds (6), and chlorinated organic compounds (7). The type strain of this species, *R. aetherivorans* JCM 14343 (originally named strain 10bc312), was originally isolated from petrochemical biotreater sludge as a methyl *tert*-butyl ether (MTBE)-degrading strain (3). Our recent study revealed that this strain is also capable of degrading recalcitrant cyclic ethers, such as 1,4-dioxane and tetrahydrofuran (4). To further understand the genetic basis of the degradation of ethers and other abilities, genome sequence analysis of *R. aetherivorans* JCM 14343^T^ was performed.

The sequenced strain, *R. aetherivorans* JCM 14343^T^, was provided by Riken BRC through the National Bio-Resource Project of the Ministry of Education, Culture, Sports, Science, and Technology (MEXT), Japan. After cultivation in malt extract-glucose-yeast extract (MGY) medium (8), total genomic DNA (gDNA) of *R. aetherivorans* JCM 14343^T^ was extracted using a NucleoSpin tissue kit (Macherey-Nagel, Düren, Germany) and purified with ethanol precipitation and using the NucleoSpin gDNA cleanup kit (Macherey-Nagel). The purified DNA was fragmented and prepared into a sequence library using the TruSeq Nano DNA LT library preparation kit (Illumina, San Diego, CA, USA). This library was sequenced with 101-bp paired-end sequencing using the Illumina HiSeq 2500 system, which produced 22,532,896 reads with a yield of 2,276 Mbp. Unless otherwise noted, default parameters were used for all software tools. From the obtained sequence reads, the adapter sequences were trimmed using Cutadapt v. 1.1, and low-quality-value regions were further trimmed using Trimmomatic v. 0.32 (http://www.usadellab.org/cms/?page=trimmomatic). The trimmed reads were assembled onto the draft genome sequence using Velvet v. 1.2.08 (https://www.ebi.ac.uk/~zerbino/velvet/). The draft genome sequence had a total length of 6,442,200 bp with a G+C content of 70.2% and a genome coverage of 353× and is based on 265 contigs with an *N*_50_ value of 159,427 bp and a maximum contig length of 504,978 bp.

The genome was annotated with Rapid Annotations using Subsystems Technology (9). Totals of 5,988 coding sequences, 49 tRNAs, and 6 rRNAs were predicted. The genome of *R. aetherivorans* JCM 14343^T^ contains alkane hydroxylase genes (an alkane monooxygenase gene, two rubredoxin genes, and a rubredoxin reductase gene) identical to those of *Rhodococcus* spp. The alkane hydroxylase is involved in the initial MTBE oxidation (10). Additionally, the genome contains propane monooxygenase genes (a type of soluble di-iron monooxygenase), which are similar to those of *Mycobacterium* spp. and are considered to be associated with the initial hydroxylation of cyclic ethers (e.g., 1,4-dioxane and tetrahydrofuran), leading to the cleavage of their high-energy C-O bond (11).

### Data availability.

The whole-genome shotgun project of *R. aetherivorans* JCM 14343^T^ has been deposited at DDBJ/EMBL/GenBank under the accession number BLAH00000000. The version described in this paper is the first version, BLAH01000000, and consists of sequences BLAH01000001 to BLAH01000265. The raw sequencing reads were deposited under accession number DRR201558. The BioProject and BioSample numbers are PRJDB8788 and SAMD00186790, respectively, and the assembled and annotated set of contigs is publicly available under assembly accession number GCA_009176285.
